# Exploring the Therapeutic Potentials of iNKT Cells for Anti-HBV Treatment

**DOI:** 10.3390/pathogens3030563

**Published:** 2014-07-03

**Authors:** Agnieszka Lawrenczyk, Seil Kim, Xiangshu Wen, Ran Xiong, Weiming Yuan

**Affiliations:** Department of Molecular Microbiology and Immunology, Keck School of Medicine, University of Southern California, Los Angeles, CA 90033, USA; E-Mails: alawrenczyk@calibr.org (A.L.); seilkim@usc.edu (S.K.); xiangshw@usc.edu (X.W.); rxiong@usc.edu (R.X.)

**Keywords:** HBV, NKT, humanized mice, antivirals, immunotherapy

## Abstract

CD1d-restricted invariant NKT (iNKT) cells are a group of innate-like regulatory T cells that recognize lipid antigens. Both mouse modeling experiments and human clinical studies have suggested a key role for iNKT cells in anti-HBV immunity and these potent T cells can be explored as a novel therapeutic target for anti-HBV treatment. We aim to humanize mice in the CD1d/iNKT cell lipid presentation system and provide new research tools for identifying novel anti-HBV agents.

## 1. Introduction

Hepatotropic viruses are known as “silent killers”, as they can manifest disease without visible symptoms. Hepatitis B virus (HBV) is one of the major public health problems worldwide and among the two billion people infected with HBV [1], an estimated 350 million are chronically infected with the virus [2]. Chronic HBV infection can lead to liver fibrosis, cirrhosis and hepatocellular carcinoma (HCC) [1], and kills approximately one million people each year [2]. Nearly 53% of HCC cases are HBV related [3]. Chronic HBV carriers include those whose blood contains HBV viral particles six months after infection [4]. The adult immune system is usually able to clear the initial HBV infection, but in infected children, who mostly contract HBV via vertical transmission from an infected mother, the clearance is not as efficient. In fact, 90% of children infected with HBV become chronically infected [1,5] compared to only 5%–10% of adults [3]. Therapy for chronic hepatitis B (CHB) includes the use of nucleotide/nucleoside analogues (NAs) and Type I interferons (IFN-α). However, these treatments are not always available, and are only effective in controlling the infection but not eliminating the virus. IFN-α therapy has many side effects and is expensive. While newer NAs are less vulnerable to the development of drug resistance compared to previous NA drugs, complete clearance of viral infection is not achieved under this regimen [6]. Therefore, new clinically viable therapeutic agents for hepatitis B infection are urgently needed.

HBV is a 3.2 Kb, partially double-stranded DNA enveloped virus that infects hepatocytes. HBV is not directly cytopathic [7] and upon infection it remains latent within the hepatocyte [8]. HBV viral proteins alter gene expression and induce oncogenesis by promoting cell proliferation, tissue invasion and metastases as well as resistance to growth inhibition and apoptosis. Although integration of HBV genome to host chromosome is not essential for HBV life cycle, it can be observed. In HBV life cycle,one of the viral genes coding for the HBx protein potentially contributes to hepatocytes malignancy and transformation [1]. During chronic exposure to HBV, there is persistent inflammation accompanied by liver damage and cell death. These factors give rise to chronic liver disease [7].

## 2. Immune Responses to HBV

The liver plays a key role in many physiological processes and is continuously exposed to toxins, intestinal flora and dietary proteins [9]. Therefore, under normal conditions, resident liver sinusoidal endothelial cells (LSEC) and Kupffer cells secrete IL-10 and TGF-β, maintaining a tolerogenic environment and dampening inflammatory responses to foreign invaders such as HBV [9,10]. The immune response to HBV can be separated into three sequential phases: immune tolerance, immune breakthrough, and immune clearance [8]. During immune tolerance, HBV further suppresses anti-viral immune responses and exploits the liver’s inefficiency in CD8^+^ cytotoxic T lymphocytes (CTL) activation, allowing it to reach an immune-tolerant state through the induction of T cell anergy and deletion of virus-specific T cell [5]. Resident LSEC and Kupffer cells increase their production of the anti-inflammatory cytokines IL-10 and TGF-β and reduce their expression of Toll-like Receptors (TLRs), leading to the inactivation of innate immunity [8]. Specifically, one viral protein, hepatitis B e antigen (HBeAg), can reduce the expression of TLR2 in hepatocytes, Kupffer cells and monocytes [11]. More importantly, there is an overall lack of Type I IFN induction, which is critical in the initiation of the antiviral immune responses. Patients with acute HBV infection have undetectable levels of these pro-inflammatory cytokines for up to 30 days post infection [12], demonstrating the lack of immune activation during the tolerance phase of HBV infection. Previously, it was believed that this lack of immune activation was due to the lack of IFN-α/β induction [13]. However, it has recently been shown that this lack of activation is instead caused by the active suppression of the innate immune system by HBV viral proteins [14]. Further suppression is mediated by the hepatic dendritic cells (DCs), which secrete IL-10 and, thereby, contribute to the activation of CD4^+^CD25^+^FoxP3^+^ T regulatory cells (Tregs). Tregs function in T cell inhibition and further suppress the activation of adaptive immunity. Additionally, up-regulation of co-inhibitory Programmed Death receptor-1 (PD-1) in CD8^+^ CTLS by Tregs leads to the exhaustion of CD8^+^ CTLs [7]. During the tolerance phase, there is no liver damage, but since antiviral mechanisms have been halted, HBV continues to replicate within the hepatocytes [7,8].

The factors triggering the transition from the immune tolerance stage to the immune breakthrough stage are not well understood. During the immune breakthrough phase, there is a strong increase in the production of IL-12, IL-18, and IFN-α, concomitant with a decrease in IL-10 production. This altered cytokine profile leads to the activation of innate immunity, recruiting cells that participate in hepatic cytotoxicity and the control of HBV replication. Specifically, infected hepatocytes produce IFN-α/β, modulating the activities of antigen presenting cells (APCs), including DCs and Kupffer cells. In addition, Natural Killer (NK) and Natural Killer T (NKT) cells are activated in response to IFN-α/β [8]. NK cells are able to detect HBV infected hepatocytes based their expression of stress ligands and lack of MHC class I molecules [15]. However, at this stage, the increased cytokine production and the activation of NK and NKT cells are not able to achieve complete control of the HBV infection. To achieve complete clearance, HBV-specific CD8^+^ T cells are essential [16]. Importantly, non-cytolytic mechanisms mediated by cytokines are crucial for HBV DNA eradiation and viral clearance. Guidotti *et al.* reported that during acute virus infection in Chimpanzees, HBV DNA clearance was achieved without the destruction of infected hepatocytes, suggesting that control of infection is mediated through non-cytolytic processes. This inhibition occurs by the release of IFN-γ and TNF-α cytokines by virus-specific CD8^+^ T cells, initiating a cascade of events which inhibit the gene expression and replication of HBV [17].

Although HBV-specific CD8^+^ T cells are essential in viral clearance, their function may be limited because of liver protective mechanisms. These protective mechanisms include Tregs, which limit CD8^+^ T cell activation and proliferation and up-regulate PD-1 expression on CD8^+^ T cells [7,8]. How the antiviral arm and protective arm of the liver immune system interact with each other likely determines the outcome of the HBV infection. In 90% of adults, HBV infections are self-resolving, and viral eradication is achieved at the immune clearance stage of infection. During immune clearance, there is a profound up-regulation of IL-12, IL-18, and IFN-α, leading to the activation of DC, NK, and T helper 17 (Th17) cells and monocyte infiltration into the liver [8]. IL-12 and TNF-α stimulate IFN-γ secretion and induce the down-regulation of PD-1 and the proliferation of CD8^+^ T cells [14]. During this stage there is successful differentiation and maturation of memory T cells. The activation of adaptive immunity is able to decrease hepatitis B surface antigen (HBsAg) and lead to the resolution of the infection [18].

In cases where the immune clearance stage is not reached, HBV establishes chronic infection. The failure of the immune system to clear the virus leads to chronic liver inflammation. In the pathogenesis of CHB there is evidence of progressive inflammatory liver damage and viral persistence [7]. Chronic inflammation is accompanied by simultaneous tissue destruction and repair [19]. The continuous recruitment and relentless activity of inflammatory cells ultimately leads to hepatic cell death [8] and liver damage [20]. Beginning the cycle of their continuous activity, macrophages, lymphocytes, and other mononuclear cells, constantly infiltrate the site of injury, leading to further tissue damage.

Since HBV is a non-cytopathic virus, the progression of chronic hepatitis is attributed to a weakened host response against viral infection. Primarily, HBV persistence is believed to be a direct result of the failure of HBV-specific CD8^+^ and CD4^+^ T cells to eliminate HBV [16]. During CHB infections there is a marked exhaustion of CD8^+^ CTLs, the main line of defense against HBV. CTLs are critical in the elimination of pathogens and secrete cytolytic mediators, including granzyme B and perforin, as well as IFN-γ and TNF-α [21]. Notably, patients with CHB display many features of T cell exhaustion and anergy [22]. Furthermore, they have ineffective CD4^+^ T cell priming during the early stages of infection, decreasing the CD8^+^ CTL potential to mount an adequate antiviral response [16]. Accordingly, these patients have progressively lower CTL frequency and show functional impairment of T helper cells, both in the peripheral blood and the liver [8]. Anergic T cells express high levels of PD-1 [7] and fail to respond to previously encountered antigenic stimuli from functional antigen-presenting cells [22]. The loss of T cell function is a gradual process in which T cells become exhausted through different stages of functional impairment [5]. Specifically, CD8^+^ CTLs from HBV-infected patients lack the ability to proliferate and produce cytokines, such as IL-2 and IFN-γ, and present an overall reduction in their cytotoxic activity. This CTL dysfunction is attributed to the exceptionally high levels of viral antigens, HBsAg and HBeAg [6], and is related to the quantity of viral replication [15,23]. Furthermore, the overall production of IL-10 and TGF-β by hepatic DCs induces the immunosuppressive function of Tregs, disrupting the ability of virus specific T cells to proliferate [6]. Increased amounts of CD4^+^CD25^+^ Tregs are found in CHB patients and are associated with elevated viral titer and HBeAg viral protein levels [24]. Much like PD-1, Tim-3 is also a negative regulator of T cell function. Tim-3 is primarily involved in the induction of tolerance and the suppression of the T helper 1 (Th1) response [25]. Tim-3 is up-regulated on CD4^+^ and CD8^+^ T cells [26] and recently has been shown to be up-regulated on monocytes and CD3^+^/CD16^+^/CD56^+^ NKT-like cells isolated from peripheral blood mononuclear cells (PBMCs) in CHB patients [27]. Thus, CHB patients have multiple defects in adaptive immunity, particularly T cell responses.

The innate immune system also greatly contributes to the pathogenesis of CHB. The function of NK cells is determined by the integration of stimulatory and inhibitory signals received from the environment. The production of IL-10 by hepatic DCs [14] causes NK cells to produce insufficient amounts of IFN-γ and skewed towards cytotoxicity, which ultimately contributes to viral persistence and liver damage [7]. Moreover, NK cells in CHB patients induce the expression of TNF-Related Apoptosis Ligand (TRAIL) on hepatocytes [7], further contributing to liver injury. In addition, TLRs, the major receptors involved in pathogen recognition, are also repressed during the CHB infection [8], leading to defective cell signaling and decreased potential to eradicate the viruses.

## 3. NKT Cells in Anti-HBV Immune Responses

NKT cells are an unconventional subset of T cells that are stimulated by lipid antigens and bridge innate and adaptive immunity [28]. Invariant NKT (iNKT) cells are a unique subset of NKT cells, defined by Vα24Jα18 TCRα in humans and Vα14Jα18 TCRα in mice, and recognize lipids presented by the antigen presentation molecule, CD1d. Invariant, or Type I NKT cells can be activated by CD1d-presented α-galactosylceramide (α-GalCer), a glycolipid derived from a marine sponge [28]. On the other hand, Type II NKT cells have a diverse TCR repertoireand are stimulated by CD1d-presented myelin-derived lipid, sulfatide [29]. Upon activation, iNKT cells secret large amount of both Th1 and T helper 2 (Th2) cytokines and play key regulatory roles in antimicrobial immunity, transplant rejection, allergic responses, autoimmunity, and cancer [28].

**Figure 1:**
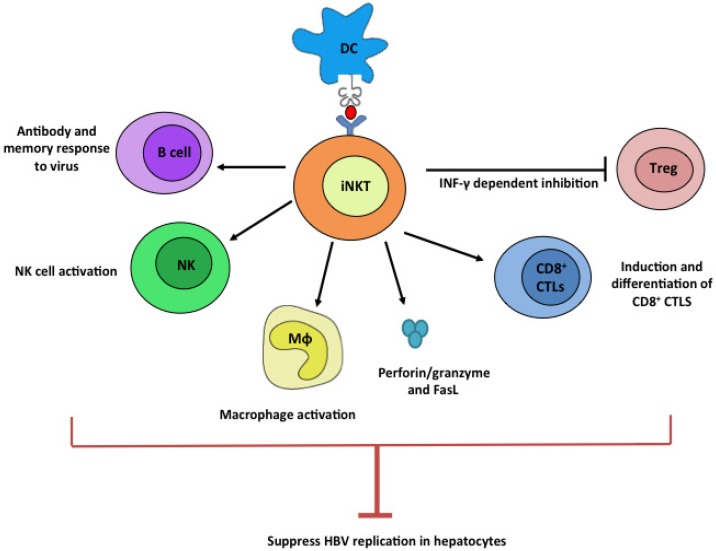
Potential anti-HBV mechanism by iNKT cells. Upon ligation with stimulatory lipid ligands presented by dendritic cells, iNKT cells potently stimulate B, NK, Mϕ, and CD8^+^ CTL cells while suppressing Treg cells. These plethoric functions of iNKT cells can all contribute to efficient suppression of HBV viruses.

iNKT cells play a central role in the regulation of the liver environment (Figure 1). One of the major functions of iNKT cells is the regulation of fibrosis through the modulation of T helper cell polarization. Th2 cytokines, including IL-4 and IL-13, have pro-fibrotic activity and up-regulate genes that promote wound healing and fibrosis. On the other hand, Th1 cytokines, such as IFN-γ, show no fibrotic activity and can attenuate tissue fibrosis [19]. Animal models of HBV have revealed that NKT cells play contrasting roles in tissue damage and fibrosis [30]. Depending on the mode of activation, they can function as pro-fibrotic or anti-fibrotic mediators by secreting Th2 (IL-4 and IL-13) or Th1 (IFN-γ) cytokines, respectively [31]. In a carbon tetrachloride (CCl_4_) model of acute liver injury in wild-type (WT) mice, iNKT cells were able to suppress the activation of hepatic stellate cells (HSC), the extracellular matrix-producing cells of the liver [30]. Interestingly, it was shown that iNKT cells activated by endogenous lipids have a protective role in liver fibrosis, while α-GalCer-activated iNKT cells cause acute liver damage and fibrosis [30]. The direction of T helper cell polarization is determined by the affinity of interaction between the TCR and the lipid presented by CD1d molecule. Lipids with weaker affinities to CD1d and TCR, such as OCH, induce a Th2 polarity, while stronger interactions, such as α-GalCer, favor Th1 polarization [32]. This polarization can also be altered by the regulation of co-stimulatory signals [33]. The differential effects of iNKT cell stimulation on liver pathology observed in different reports are likely due to the differences in the mode how iNKT cells are stimulated by lipid ligands with either weak strong affinities to CD1d and iNKT TCR.

The characterization of iNKT cells in a CHB infection has not yet been fully investigated due to technical limitations such as the restricted host range of HBV and the low frequency of blood iNKT cells in available patient blood [34]. HBV infection is restricted to humans and chimpanzees [4]. While woodchucks and Peking ducks are also hosts of HBV, their immunology is poorly understood. Furthermore, HBV also lacks the ability to be successfully grown *in vitro* [2]. To build better animal models, several elegant HBV transgenic mouse models have been generated to facilitate the understanding of this disease [16,31,35,36]. In the HBV transgenic mouse model used in our study, a DNA fragment containing a HBV genome 1.3 times of the regular HBV genome size was used to generate the transgenic mice [35]. The larger genome is required to generate a 3.5 Kb RNA intermediate of HBV replication to produce high titer HBV infection in the liver and kidneys, while shorter fragments produce low viral titer and the infection is limited to the kidneys [36]. The HBV genome contains the C, S, P, and X genes coding for precore/core proteins, envelope proteins (surface antigens), viral polymerase, and X protein, respectively. These DNA fragments were injected into fertilized mouse embryos and the resultant mice showed efficient HBV replication [35].

HBV transgenic mouse models present evidence for NKT/HBV crosstalk. In these models, α-GalCer-activated iNKT cells are able to suppress HBV replication and activate NK cells, leading to potent cytokine and nitric oxide production. iNKT cell activity is reduced in IFN-α/β receptor-deficient mice, suggesting that the activation of iNKT cells by α-GalCer is at least partially mediated by IFN-α/β [37]. Type I IFNs are integral in antiviral immunity, and minimize the pathology and spread of the HBV virus [16]. α-GalCer-activated iNKT cells are able to up-regulate the Th1 response towards HBV by recruiting NK cells to the liver and stimulating IFN-γ production by NK cells. This induction appears to be long lasting because NK cell activity is still apparent even after the iNKT cell number returns to baseline [37]. In transgenic mice, HBV disappears in response to α-GalCer-induced IFN-γ production, but prior to T cell influx to the liver demonstrating the potential of iNKT cells to induce T cell-independent antiviral activity [37].

iNKT cells are potent activators of the immune system and their activation promotes the loss of tolerance to HBV-specific CD8^+^ T cell antigens in mouse models (Figure 1, [21]). Moreover, iNKT cell activation by α-GalCer induces IL-2-dependent activation of HBsAg-specific CTLs in HBsAg transgenic mice and enhances the expression of IFN-γ, TNF-α, IL-2, and IL-4, all of which affect CTL induction and proliferation [21]. Upon activation, iNKT cells express high levels of granzyme B, perforin, and FasL [38], thereby mediating the direct killing of HBV infected cells. iNKT activation also recruits neutrophils, myeloid dendritic cells, macrophages, B cells, CD4^+^ and CD8^+^ T cells [38], aiding in viral clearance. Recently, studies have demonstrated that iNKT cells are able to regulate Treg activity [39], which is critical for HBV pathogenesis based on the high prevalence of Tregs in the liver during HBV infection. IL-2 production from CD4^+^ NKT cells is able to modestly induce the proliferation of Tregs. Tregs are able to suppress iNKT cells through contact dependent inhibition [39]. Importantly, Oh *et al.* found that through production of IFN-γ, iNKT cells are able to inhibit the induction of Tregs [40]. Clinically, offsetting Treg activity can provide an opportunity for adaptive immunity to inhibit HBV replication and clear the infection. Furthermore, activation of iNKT cells in mice was shown to enhance B cell memory and improve antibody titer [41]. This could potentially improve the antiviral responses by increasing the anti-HBV antibodies. In addition, the activation of iNKT cells can stimulate the induction and differentiation of CD8^+^ T cells, prime the adaptive immune system through the up-regulation of MHC class I and MHC class II antigen presenting molecules and stimulate NK cells to secrete IFN-γ (Figure 1, [38]), significantly improving the prognosis of CHB.

## 4. iNKT Cell-Based Immunotherapy against HBV in Humans

HBVtg mouse models have demonstrated that iNKT cells can play a potent role in the immunity against hepatitis B [21,37]. The striking potency of α-GalCer in inhibiting HBV replication in transgenic mice has raised great interest in using this lipid for HBV immune-therapy. Unlike highly polymorphic MHC class I and II molecules, CD1d is mono-allelic therefore lipid drug candidates targeting CD1d and iNKT cells can be applicable to populations of diverse background. Lipids are also generally of low toxicity to humans. Furthermore, because iNKT recognize lipid antigens rather than viral peptide antigens, they can not be deleted during chronic infection [37]. However, human clinical trials have failed to show a sustained and consistent decrease of HBV DNA post α-GalCer administration [42,43]. Thus, there is a sheer discrepancy between the anti-HBV roles of iNKT-targeting lipids in mice and humans. Similarly, α-GalCer has shown highly potent anti-tumor activity in mouse models, while human clinical trials have shown limited success [42].

Discrepancies in anti-HBV function of iNKT cells between mouse and human models can be explained, at least partially, by subtle yet important differences between the mouse and human CD1d/NKT antigen presentation systems. The mouse and human CD1d molecules share a high degree of conservation in three-dimensional structures and display approximately 65% amino acid sequence homology [44]. Despite these similarities, the crystal structure of the CD1d-α-GalCer/iNKT TCR complexes revealed significant differences in the orientation and conformation of the CD1d between the mouse and human systems upon CD1d-α-GalCer binding [32]. For example, hCD1d molecule in particular has a bulky Trp153 residue (as opposed to Glycine155 in mCD1d) in its interaction interface with α-GalCer that causes a shift of galactose head group and likely decreases hCD1d interaction with α-GalCer [32,45]. These subtle conformational and orientation differences can significantly change the CD1d-α-GalCer affinity to the iNKT TCR [32,44], resulting in different response potencies and/or alteration in Th1 and Th2 polarization [44]. Additional differences in the binding interface of the mouse and human CD1d molecules to their cognate iNKT TCRs can also impact the affinity of CD1d-α-GalCer complexes to their respective TCRs [32,45]. All these differences are expected to affect the presentation of CD1d presentation of α-GalCer and lead to different responses of iNKT cells in human and mouse models.

The iNKT cell populations also differ greatly between human and mouse immune systems (Table 1). The human iNKT TCR consists of an invariant Vα24 chain paired to Vβ11, while murine iNKT cells express Vα14 paired to either Vβ8, Vβ7, or Vβ2 [28]. Mouse Vβ8 TCRβ is the closest homolog of human Vβ11 TCRβ chain and has the greatest compatibility with hCD1d. Indeed, a humanized CD1d mouse knock-in model showed that expression of hCD1d leads to preferential selection of Vβ8 iNKT cells [42]. One major difference between human and mouse iNKT cells lies in their tissue abundance. While the iNKT cells comprise 20%–30% of liver monocytes in mice, human liver iNKT cells are only approximately one tenth as abundant. iNKT cells can be further classified based on their CD4 and CD8 co-receptor expression [28,38]; human iNKT cells include CD4^+^, CD8^+^, and CD4^−^CD8^−^ double negative (DN) subsets, while mouse iNKT cells are only composed of CD4^+^ and DN subsets. Humans possess more abundant DN iNKT cells as compared to more abundant CD4^+^ iNKT cells in mice. These two iNKT subsets have distinct physiological roles [28,33]. Upon activation, CD4^+^ iNKT cells display a regulatory phenotype and produce both Th1 and Th2 cytokines, while DN iNKT cells preferentially produce Th1 cytokines [28,38]. All these differences are expected to substantially impact the interaction of HBV with the liver immune system and result in differences in HBV pathogenesis in human patients *versus* that in mice.

**Table 1:** Differences in iNKT cells between human and mice.

	Mice	Human
**TCR usage**	Vα14/Vβ8, 7, 2	Vα24/Vβ11
**Frequency in tissues**	Highest in liver (~20%–30%)	Highest in liver (~1%–2%)*
**Subsets**	CD4^+^, DN	CD4^+^, DN, CD8^+^
**Composition**	CD4^+^ > DN	DN > CD4^+^
**Development and maturation**	Develop and mature in thymus	Mature in peripheryCD4^−^ subsets develop in periphery

*One report showed 10% iNKT cell frequency in human omentum [34].

**Figure 2:**
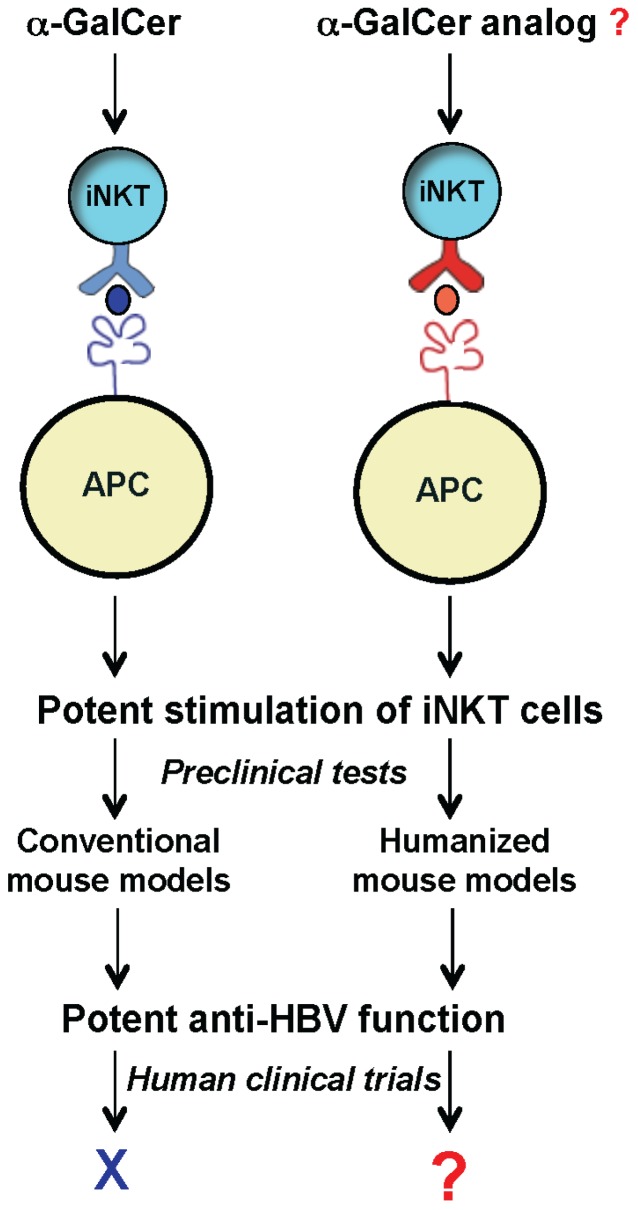
Exploring the therapeutic potential of iNKT cells for anti-HBV treatment using humanized mouse models. New humanized mouse models (with mouse CD1d and iNKT TCR replaced with human counterparts) can be used as a platform to identify and test novel glycolipids, such as α-GalCer analogs for inhibiting HBV in human clinics.

To develop a mouse model that more closely recapitulates the human CD1d/NKT cell system, we generated humanized mice harboring human components of this antigen presentation system. Recently we reported the first human CD1d-knock in (hCD1d-KI) mouse [42]. In this model, the mCD1d1 gene was replaced with the hCD1d gene. CD1d1 is responsible for the murine NKT cell development, and the replacement with the hCD1d construct led the iNKT cell development into a human-like iNKT cell phenotype including CD4 and NK1.1 expression [42]. Our model showed substantial differences in the abundance of iNKT cells in major immune organs compared to that in wild-type mice. The frequency of hepatic iNKT cells is 1%–2%, resembling the abundance in humans [42]. With similar abundance and phenotypes to that of human iNKT cells, this model provides a unique opportunity to identify novel lipid ligands that can potently stimulate the iNKT cells and inhibit HBV replication in human patients (Figure 2).

To build a humanized HBVtg mouse model, we have introduced the HBV transgene into our CD1d-humanized mouse. The novel HBVtg/hCD1d-KI model showed a statistically significant decrease of iNKT cells [46]. The number of iNKT cells in the liver dropped from an average of 1%–2% in healthy mice to approximately 0.6% in our HBV model [46]. This decline of the iNKT cell population is consistent with a clinical study performed by Jiang *et al.*, where CHB patients also displayed a decreased population of circulating iNKT cells [47]. *Ex vivo* investigation revealed that these lower frequencies of iNKT cells showed no impairment in their ability to produce IFN-γ, suggesting that the remaining iNKT cells were still functional. Antiviral treatment with Telbivudine could restore the levels of circulating iNKT cells, particularly CD4^−^ iNKT cells, in CHB patients [48].

Human CD4^+^ iNKT subpopulations also express their own unique set of receptors and display distinct functions. CD4^+^ iNKT cells tend to express increased CD62L compared to their CD4^−^ iNKT counterpart, which expresses high levels of CD11a. Based on this display of receptors, CD4^−^ iNKT cells exhibit a tissue-infiltrating phenotype as opposed to the lymph node homing phenotype in CD4^+^ iNKT cells. CD4^−^ iNKT cells also express NKG2D receptor [28], which recognizes stress ligands and is expressed on both NK and iNKT cells. NKG2D receptor has been directly implicated in HBV pathogenesis [49,50]. Blocking of the NKG2D receptor has been shown to decrease the pathogenic effects caused by HBV [49,50]. Mouse models have demonstrated that many diseases impact the CD4^+^/CD4^−^ ratio of iNKT cells [41,51]. Indeed, our model not only revealed a profound decrease in iNKT cells but also showed altered proportions of CD4^+^ and CD4^−^ iNKT cell populations, with a higher CD4^−^/CD4^+^ ratio [46]. This implies that HBV may lead hepatic iNKT cells to become more Th1 polarized.

A common feature in CHB is CD8^+^ T cell anergy. The up-regulation of PD-1 leads to the exhaustion of CD8^+^ CTLs [2,8,16]. Our mouse model indeed revealed that there is a significant up-regulation of PD-1 on conventional T cells, which is restricted to the CD8^+^ population [46]. Interestingly, we did not detect PD-1 expression in the iNKT cells in the HBV transgenic mice [46], consistent with the functional activity of the iNKT cells shown in human CHB patients [47,49] and suggesting a great potential of exploiting the iNKT cells to boost the immunity against HBV. With the availability of humanized HBVtg mice, it will be exciting now to examine and evaluate the anti-HBV properties of novel glycolipid ligands of iNKT cells in these new mouse models (Figure 2).

## 5. Future Perspectives

This review is focused on the therapeutic potential of exogenous lipid ligands that trigger an iNKT cell-dependent anti-HBV immune response and the development of a novel humanized mouse model for the identification and pre-clinical evaluation of the candidate molecules. Future mechanistic studies will be needed to thoroughly examine the immune responses of these potentially potent anti-HBV lipid drug candidates in a humanized immune system setting before we can move into clinical trials. We have not discussed the potential of stimulating endogenous lipid presentation by CD1d to mobilize NKT cells for anti-HBV therapy. Animal models have shown that β-glycosphingolipids can act as vaccine adjuvants, influence NKT frequencies and function as well as stimulate NKT cell-mediated antiviral immunity, further demonstrating possible therapeutic uses of NKT cells [52]. On the other hand, precise modulation of Type I *versus* Type II NKT cells will prove to be critical for optimizing NKT cell-based anti-HBV therapy. It was recently demonstrated that HBV infection of hepatocytes can lead to the activation of both Type I and Type II NKT cells, through production of endogenous endoplasmic reticulum (ER)-associated antigenic lipids [53]. Specifically, it was showed that invariant Type II NKT cells are activated upon HBV infection by CD1d presentation of an antigenic CD1d ligand, lysophosphatidylethanolamine (lysoPE), leading to a cytokine-dependent activation of NKT cells through the presumable activation of liver DCs and macrophages [53]. It is foreseeable that more exciting research will be coming in these areas as well.

## 6. Conclusive Remarks

Considering the discrepancies in the anti-HBV potency of iNKT cell-targeting lipid ligands in conventional mouse models and humans, humanized HBVtg mouse models can serve as a new platform for identifying novel anti-HBV therapeutic agents. These new mouse models can bridge the gaps created by the subtle differences in lipid-presenting properties of hCD1d and mCD1d molecules and the overt phenotypic differences between human and mouse iNKT cells and ultimately facilitate the discovery of novel Th1-polarized anti-HBV lipid ligands of iNKT cells, a promising yet little explored target for anti-HBV therapy.
